# Dietary sugar intake and risk of depression: findings from the TwinsUK cohort

**DOI:** 10.1016/j.nupsyc.2026.100025

**Published:** 2026

**Authors:** Idrees Mughal, Zeeshan Mughal, Khaylen Mistry, Noor Dhakal, Faraz Mughal

**Affiliations:** a University Hospitals Birmingham NHS Foundation Trust, Birmingham, UK; b Abbey Medical Centre, 42 Station Road, Kenilworth, CV8 1JD, UK; c Department of Dermatology, Norfolk & Norwich University Hospital, Colney Ln, Norwich, NR4 7UY, UK; d Department of Cardiology, Royal Berkshire Hospital, 3-5 Craven Rd, Reading, RG1 5LF, UK; e Oxford Primary Care Clinical Trials Unit, Nuffield Department of Primary Care Health Sciences, University of Oxford, OX2 6GG, UK; f School of Medicine, Keele University, ST5 5GB, UK

**Keywords:** Depression, Sugar intake, Dietary sugars, Nutritional psychiatry, Cohort study

## Abstract

**Background:**

High intakes of sugars have been linked to depression, but few studies have accounted for both sugar and fat subtypes, which is important because many high-sugar foods also contain substantial fat. This study examined the association between dietary sugar intake and depression risk while accounting for multiple fat subtypes.

**Methods:**

Using data from the UK TwinsUK cohort (cross-sectional analyses: n = 3029 in 2002 and n = 3452 in 2017; longitudinal analysis: n = 1485) associations between sugar intake and depression risk were examined at two time points and longitudinally. Depression was assessed using the Hospital Anxiety and Depression Scale (HADS) in 2002 and 2017, and dietary intake was estimated using a validated 120-item food frequency questionnaire at corresponding periods. Cross-sectional analyses used binary logistic regression to model HADS-defined depression cases, and longitudinal analyses used linear regression to examine change in HADS outcomes. Models were adjusted for total sugars and sugar subtypes, fat subtypes, age, body mass index, sex, Index of Multiple Deprivation and alcohol intake.

**Results:**

Higher total sugar intake was associated with greater odds of HADS-defined depression in 2002 (odds ratio (OR) 1.34 per 100 g, 95% confidence interval (CI) 1.07 to 1.68) and 2017 (OR 1.41 per 100 g, 95% CI 1.21 to 1.65). In longitudinal analyses, increasing sugar intake was associated with higher HADS scores over follow-up (b 0.63, 95% CI 0.10 to 0.99).

**Conclusions:**

Higher sugar intake was associated with higher depressive symptom scores, independent of fat subtypes, and may reflect broader dietary patterns including higher consumption of ultra-processed foods. Further randomised controlled trials are needed to determine whether reducing dietary sugar intake can improve depressive symptoms.

## Highlights

• Higher dietary sugar intake was associated with greater odds of HADS-defined depression.
• Associations were observed after adjustment for dietary fat subtypes and other covariates.
• Longitudinal analyses showed higher sugar intake was associated with higher HADS scores over follow-up.
• Findings support further investigation into dietary patterns and depressive symptoms.

## Abbreviations

BDNF: brain-derived neurotrophic factor
BMI: body mass index
CI: confidence interval
CMD: common mental disorder
FFQ: food frequency questionnaire
GI: glycaemic index
GL: glycaemic load
HADS: Hospital Anxiety and Depression Scale
HPA: hypothalamic–pituitary–adrenal
IMD: Index of Multiple Deprivation
OR: odds ratio
RCT: randomised controlled trial
SD: standard deviation
UK: United Kingdom

## 1 Introduction

The oral consumption of sugars has been identified as a modifiable risk factor in diseases such as obesity, diabetes and cardiovascular disease [1]. In the United Kingdom (UK), adults consume on average approximately double the recommended level of added sugars (5% of energy intake) [1]. Furthermore, the proportion of ultra-processed foods in the diet is increasing in countries such as the United States, making up >50% of their energy intake [2] and global consumption of ultra-processed foods continues to rise as more countries adopt westernised dietary patterns [3]. Meanwhile, depression is predicted to become the leading cause of disability in high-income countries by the year 2030 [4]. There is sufficient evidence that poor dietary quality and sugar intake are linked to worsening depression outcomes [[5], [6], [7]].

Several cross-sectional studies have concluded that higher sugar intake is associated with a greater risk of depression [6,8]. Furthermore, prospective cohort studies have investigated the link between sugary foods and drinks with depression [5,9,10]. Knüppel et al. found that men in the highest tertile of sugar intake from sweet foods and beverages had a 23% increased odds of Common Mental Disorder (CMD) (reporting ≥5 symptoms on the General Health Questionnaire) after 5 years (OR = 1.23, 95% CI: 1.02, 1.48) [9]. Another study found the OR for CMD comparing ≥4 cups of sweetened beverage to none was 1.30 (95% CI: 1.17, 1.44 p < 0.01) [5]. A recent meta-analysis of thirty-eight cross-sectional, cohort and case-control studies, including over one million participants showed that the highest versus lowest category of sugar intake increased the risk of depression by 21% (OR = 1.21, 95% CI: 1.14, 1.27) [11]. Although these studies found an increased risk of depression with a higher consumption of sugars, the majority of these failed to adjust for dietary complexities such as individual fat sub-types. Many processed and sweet foods contain high levels of both sugars and fats and current evidence indicates that certain types of fats in food can influence mood states [[12], [13], [14], [15]]. Saturated and trans-unsaturated fatty acids show positive associations with mood disorders [14]. Pro-inflammatory cytokines expressed during the consumption of saturated and trans-unsaturated fatty acids are hypothesised to influence mental health [16]. Furthermore, mono and polyunsaturated fatty acid intake is associated with improved mental health outcomes [[12], [13], [14]].

There are several potential mechanisms for an association of sugar intake and subsequent depression risk: influence on the growth factor, brain-derived neurotrophic factor (BDNF) [17] and an increase in systemic inflammation [18] have both been discussed as part of the pathogenesis of depression. Sugar intake and low mood are also linked by fluctuating blood sugar levels influencing neurotransmitters [19].

The aim of this study was to investigate whether total sugar intake and its sub-types are associated with depressive symptom outcomes whilst accounting for dietary fat subtypes as potential confounders. To our knowledge, this is the only study to adjust for sugar and fat subtypes, when assessing depression risk.

## 2 Methods

### 2.1 Study cohort

The study participants were twins enrolled in the TwinsUK Cohort study, a national cohort of adult twin volunteers recruited in 1992 [20]. In total 16,976 identical and non-identical twins (19.5% male, 80.5% female) aged between 18 and 100 were recruited [21]. The TwinsUK cohort is predominantly female, which reflects the original recruitment strategy of the registry. The cohort was chosen for this analysis because of its strong dietary and mental health data [22].

The participants included in this analysis answered the Hospital Anxiety and Depression Scale (HADS) in 2002 and 2017, as well as a 120-item validated Food Frequency Questionnaire (FFQ) at two time points (1993-2001, 2014).

Participants were eligible for inclusion if they had linked FFQ and HADS data available at time points. Across all analyses, 7966 participant observations were included from the original TwinsUK cohort. These time points were selected based on the availability of linked dietary and mental health data within the TwinsUK cohort rather than predefined exposure intervals.

Of these participants, 3029 had completed FFQ1 (1993–2001) and HADS in 2002 and were included in the 2002 cross-sectional analysis. A separate subgroup of 3452 participants had completed FFQ3 (2014) and HADS in 2017 and were included in the 2017 cross-sectional analysis.

Finally, 1485 participants had complete repeated FFQ and HADS measurements across both time periods and were therefore included in the longitudinal analysis. The interval between FFQ1 and HADS assessment in 2002 varied between participants depending on timing of questionnaire completion. This approach allowed assessment of whether habitual dietary patterns preceded later depressive symptom measures.

### 2.2 Ethics approval and consent

This study was conducted according to the guidelines in the Declaration of Helsinki and all procedures involving human subjects were approved by the NHS North West – Liverpool Central Research Ethics Committee (REC reference 23/NW/0107). TwinsUK Biobank approval was renewed by the same committee (REC reference 23/NW/0106; IRAS ID 341962). Written informed consent was obtained from all subjects.

### 2.3 Assessment of sugar intake

Sugar intake was measured via the 120-item FFQ at two time points which captured habitual dietary intake over the previous year, including frequency and portion size of commonly consumed foods [23]. Sugar intake was calculated by multiplying food frequencies per day by their sugar content and portion size based on McCance and Widdowson's, The Composition of Food, 6th edition [24]. This provided daily macro and micronutrient values for each participant. This includes total sugars, and the subtypes of sugars including glucose, fructose, sucrose, lactose and maltose. This was done prior to receiving the data by the TwinsUK research team.

### 2.4 Assessment of depression

Mental health status was assessed in 2002 and 2017 via the validated HADS questionnaire, which comprises of a series of questions about somatic and psychological symptoms over the past two weeks [25]. This is scored using a 4-point Likert scale and targeted at identifying those at risk of depression and anxiety. The questionnaire is split into seven depression and seven anxiety-related questions. Participants scoring >7 on the HADS depression subscale were classified as HADS-defined depression cases, consistent with commonly used screening thresholds in the literature [25]. However, HADS is a screening instrument and does not constitute a clinical diagnosis of depression. Those with missing HADS data were handled using the ‘half rule’, where if more than half of the questions were answered, the average response score was applied to the remaining answers [26]. For cross-sectional analyses, the HADS depression subscale was analysed as a binary outcome using the established threshold of >7 to define HADS-defined depression cases versus non-cases. For longitudinal analyses, total HADS score and HADS depression sub-score were analysed as continuous variables.

### 2.5 Covariates

Potential covariates were chosen based on review of the literature and restricted to variables that were available for analysis in the cohort. Alcohol intake (g), weight (kg) and height (cm), Index of Multiple Deprivation (IMD), gender, age, ethnicity and twin zygosity data were gathered as potential covariates. Weight and height were converted into a body mass index (BMI) score for each participant, using the formula “weight(kg)/height^2^(in m^2^)”. IMD was used as ‘combined IMD’ which assumes comparison between countries (England, Scotland, Wales, Northern Ireland) is appropriate. Participants with missing weight, height, gender or age data were excluded prior to statistical analyses and final analytic sample selection (n = 12). In addition, consumption of fats and fat subtypes was classified as a potential confounder as there is evidence to suggest they play a role in the development of mental illness [27,28] and vitamin C was used as a marker of diet quality in order to distinguish between refined and natural sources of sugar [29].

### 2.6 Statistical analyses

Analysis consisted of cross-sectional models at two time points (2002 and 2017) followed by models of change. Binary outcomes were analysed (depression cases vs non-cases) for the cross-sectional analyses. Both analyses were conducted using binary logistic regression models [30]. Each variable was analysed independently, checked for correlations to other variables, then added to the adjusted models. The analyses at both time points were adjusted for gender, age, BMI, combined IMD, percentage of sugar subtypes, alcohol intake and the percentage of fat subtypes. For the longitudinal analysis for change over time, HADS and the depression sub-scores of HADS were measured as continuous variables (range of scores = 0-42 for HADS, 0-21 for depression-related questions). This was analysed using linear regression analysis, first independently (adjusting for baseline HADS), then a multivariate linear regression analysis was used to adjust for confounding to calculate beta coefficients. Longitudinal analyses were adjusted for baseline HADS and depression score, BMI, age, combined IMD, gender, alcohol intake and vitamin C.

The fat subtypes (g) were highly correlated with total sugars (g) and fats (g). Therefore, when adjusting for the sugar and fat subtypes, the percentage contribution of each sugar subtype to total sugars, and the percentage contribution of each fat subtype to total fats was used instead of values in grams. Results are presented according to 100g units. The TwinsUK is largely a Caucasian cohort, and in the sample used for analysis, >95% were Caucasian, therefore it was not appropriate to add ethnicity to the adjusted models. BMI and age were analysed as continuous variables. Combined IMD was analysed as quintiles with the first being the most deprived. 74 participants had missing IMD data. To account for this, we gave them the median combined IMD score for the cohort (as the data were not normally distributed) which was 7 (equates to the 4th quintile). All analyses were performed using IBM SPSS Statistics version 25 [31] and p < 0.05 indicated statistical significance.

## 3 Results

### 3.1 Baseline characteristics

Table 1 and Table 2 outline the baseline characteristics of participants stratified by depression status for the years 2002 and 2017, respectively. The average ages for those with and without depression were comparable in 2002, at 52 and 54 years, respectively, with over 90% of participants in both groups being female. The prevalence of depression was stable, with 9.4% of participants identified in 2002 and 9.0% in 2017. Notably, mean total sugar intake was consistently higher in the depression group at both time points, measuring 143.3g versus 133.1g in 2002, and 125.8g versus 117.3g in 2017.

**Table 1:** Participant characteristics by HADS-defined depression cases in 2002.

	Depression – Non cases (%) N = 2745 (90.6)	Depression – Cases (%) N = 284 (9.4)
**Age**, years - mean (SD)	54 (13)	52 (11)
**Sex**		
Female	2576 (93.8)	273 (96.1)
Male	169 (6.2)	11 (3.9)
**BMI** kg/m^2 - mean (SD)	24.9 (4.4)	26.3 (4.9)
**Combined Index of Multiple Deprivation** (median)	7	7
I (most deprived quintile)	153 (5.6)	28 (9.9)
II	376 (13.7)	32 (11.2)
III	544 (19.8)	58 (20.4)
IV	826 (30.1)	80 (28.2)
V (least deprived quintile)	847 (30.9)	86 (30.3)
**Total sugar intake (g)** – mean (SD)	133.1 (53.1)	143.3 (73.1)
**Total Carbohydrates (g) –** mean (SD)	270.5 (91.5)	278.5 (130.5)
**Glucose (g)** – mean (SD)	22.3 (11.8)	22.4 (14.8)
**Fructose (g)** – mean (SD)	26.8 (15.3)	25.8 (15.9)
**Sucrose (g)** – mean (SD)	53.0 (26.2)	56.0 (42.2)
**Maltose (g)** – mean (SD)	4.5 (2.8)	4.5 (2.7)
**Lactose (g)** – mean (SD)	22.5 (11.3)	23.6 (12.6)
**Alcohol (g)** – mean (SD)	10.3 (14.5)	10.6 (15.0)
**Total Fats (g)** – mean (SD)	73.9 (30.1)	78.0 (14.5)
**Monounsaturated fatty acids (g)** – mean (SD)	24.3 (10.4)	25.7 (12.1)
**Polyunsaturated fatty acids (g)** – mean (SD)	16.2 (7.0)	15.1 (6.0)
**Saturated fatty acids (g) -** mean (SD)	27.8 (12.8)	30.1 (16.7)
**Trans-unsaturated fatty acids (g)** – mean (SD)	1.9 (1.2)	2.0 (1.3)

**Table 2:** Participant characteristics by HADS-defined depression cases in 2017.

	Depression - Non Cases (%) N = 3141 (91.0)	Depression - Cases (%) N = 311 (9.0)
**Age**, years - mean (SD)	64 ( ± 13)	63 ( ± 14)
**Sex**		
Female	2833 (90.2)	293 (94.2)
Male	308 (9.8)	18 (5.8)
**BMI** kg/m^2 - mean (SD)	25.0 (4.2)	26.0 (4.6)
**Combined IMD (median)**	7	7
I (most deprived quintile)	153 (4.8)	33 (10.6)
II	392 (12.5)	50 (16.0)
III	642 (20.5)	58 (18.7)
IV	946 (30.1)	93 (29.9)
V (least deprived)	508 (32.1)	77 (24.7)
**Total sugar intake (g)** – mean (SD)	117.3 (52.3)	125.8 (60.1)
**Total Carbohydrates (g) –** mean (SD)	231.8 (100.9)	241.3 (118.0)
**Glucose (g)** – mean (SD)	22.4 (12.1)	23.0 (13.1)
**Fructose (g)** – mean (SD)	25.9 (14.1)	23.6 (12.4)
**Sucrose (g)** – mean (SD)	44.9 (24.4)	50.2 (32.8)
**Maltose (g)** – mean (SD)	3.1 (2.1)	3.4 (2.5)
**Lactose (g)** – mean (SD)	16.9 (10.9)	18.5 (13.9)
**Alcohol (g)** – mean (SD)	7.8 (11.7)	8.0 (14.4)
**Total Fats (g)** – mean (SD)	71.3 (36.2)	76.2 (39.4)
**Monounsaturated fatty acids (g)** – mean (SD)	23.4 (12.4)	25.0 (13.1)
**Polyunsaturated fatty acids (g)** – mean (SD)	16.0 (8.9)	14.6 (7.6)
**Saturated fatty acids (g)** – mean (SD)	25.2 (13.4)	27.8 (16.1)
**Trans-unsaturated fatty acids (g)** – mean (SD)	1.5 (1.1)	1.7 (1.2)

### 3.2 Cross-sectional results

Binary logistic regression analyses showed strong positive and consistent associations between total sugar intake and depression at 2002 (Table 3) and 2017 (Table 4). In 2002 the adjusted OR for depression associated with total sugar intake (per 100g) was 1.34 (95% CI: 1.07 to 1.68 p < 0.01), and in 2017 it was 1.41 (95% CI: 1.21 to 1.65 p < 0.01). These findings indicate that higher sugar intake was associated with greater odds of HADS-defined depression across the observed intake range.

**Table 3:** Results of logistic regression predicting likelihood of HADS-defined depression cases at 2002 timepoint.

	Unadjusted OR (95% CI)	P value	Adjusted^a^ OR (95% CI)	P value
**Sex**				
Female	**1.00**		**1.00**	
Male	1.63 (0.87 to 3.03)	0.12	2.16 (1.12 to 4.18)	0.02
**Age**	0.99 (0.982 to 1.00)	0.07	0.99 (0.98 to 1.00)	0.01
**BMI**	1.06 (1.04 to 1.09)	<0.01	1.07 (1.04 to 1.10)	<0.01
**Combined IMD**	1.00 (0.95 to 1.05)	0.62	1.01 (0.96 to 1.07)	0.77
**Total sugar intake (per 100g)**	1.34 (1.10 to 1.62)	<0.01	1.34 (1.07 to 1.68)	0.01
Glucose (% of total sugars)	1.02 (0.97 to 1.08)	0.42	1.01 (0.96 to 1.07)	0.62
Fructose (% of total sugars)	0.92 (0.89 to 0.96)	<0.01	0.94 (0.91 to 0.98)	0.01
Sucrose (% of total sugars)	0.98 (0.97 to 0.99)	<0.01	0.98 (0.97 to 0.99)	<0.01
Maltose (% of total sugars)	0.94 (0.89 to 1.00)	0.06	0.96 (0.90 to 1.02)	0.18
Lactose (% of total sugars)	0.98 (0.96 to 1.00)	0.01	0.98 (0.96 to 1.00)	0.03
**Alcohol (per 100g)**	1.19 (0.52 to 2.69)	0.68	1.80 (0.76 to 4.27)	0.18
**Polyunsaturated fats (% of fats)**	0.94 (0.89 to 0.98)	<0.01	0.96 (0.91 to 1.00)	0.05
**Monounsaturated fats (% of fats)**	1.04 (0.98 to 1.10)	0.22	1.06 (1.00 to 1.13)	0.05
**Saturated fats (% of fats)**	1.00 (0.99 to 1.04)	0.87	1.01 (0.97 to 1.05)	0.56
**Trans-unsaturated fats (% of fats)**	1.04 (0.96 to 1.11)	0.37	1.06 (0.98 to 1.15)	0.16

a Adjusted = adjusted for all other variables in the table.

**Table 4:** Results of logistic regression predicting likelihood of HADS-defined depression cases at 2017 timepoint.

	Unadjusted OR (95% CI)	P value	Adjusted^a^ OR (95% CI)	P value
**Sex**				
Female	**1.00**		**1.00**	
Male	0.57 (0.35 to 0.92)	0.02	0.46 (0.27 to 0.77)	<0.01
**Age**	0.99 (0.99 to 1.00)	0.21	0.99 (0.98 to 0.99)	<0.01
**BMI**	1.05 (1.03 to 1.08)	<0.01	1.05 (1.02 to 1.07)	<0.01
**Combined IMD**	0.91 (0.87 to 0.95)	<0.01	0.92 (0.88 to 0.97)	<0.01
**Total sugar intake (per 100g)**	1.37 (1.14 to 1.65)	<0.01	1.41 (1.21 to 1.65)	<0.01
Glucose (% of sugars)	1.11 (1.07 to 1.16)	<0.01	1.15 (1.10 to 1.20)	<0.01
Fructose (% of sugars)	0.84 (0.81 to 0.87)	<0.01	0.89 (0.87 to 0.91)	<0.01
Sucrose (% of sugars)	0.98 (0.97 to 1.00)	0.06	1.01 (0.99 to 1.03)	0.62
Maltose (% of sugars)	0.94 (0.87 to 1.01)	0.11	0.95 (0.88 to 1.04)	0.25
Lactose (% of sugars)	1.01 (1.00 to 1.02)	0.03	0.98 (0.97 to 0.99)	<0.05
**Alcohol (per 100g)**	1.14 (0.44 to 2.94)	0.79	1.75 (0.68 to 4.55)	0.25
**Polyunsaturated fats (% of fats)**	0.79 (0.76 to 0.83)	<0.01	0.79 (0.75 to 0.83)	<0.01
**Monounsaturated fats (% of fats)**	0.88 (0.83 to 0.94)	<0.01	0.87 (0.81 to 0.93)	<0.01
**Saturated fats (% of fats)**	0.96 (0.90 to 0.99)	<0.01	0.95 (0.89 to 0.99)	<0.01
**Trans-unsaturated fats (% of fats)**	0.92 (0.77 to 1.11)	0.41	0.90 (0.74 to 1.09)	0.28

a Adjusted = adjusted for all other variables in the table.

Higher percentage contribution of fructose and lactose to total sugars was associated with lower odds of HADS-defined depression at both time points (OR: 0.94, and 0.98 p < 0.05 in 2002, and OR: 0.89 and 0.98 p < 0.05 in 2017, respectively). Sucrose showed a similar association in 2002 only (OR: 0.98, 95% CI 0.97 to 0.99 p < 0.01). Dietary percentage of glucose was positively associated with depression at both time points, however, was only statistically significant in 2017 (OR: 1.15, 95% CI 1.10 to 1.20 p < 0.01). These subtypes were modelled as percentages of total sugars rather than absolute intake due to collinearity.

As a result, a higher proportion of fructose or lactose may reflect differences in dietary sources, such as greater consumption of fruit or dairy, rather than a direct protective biological effect of these sugars. Conversely, higher glucose proportion was positively associated with depression in 2017, which may reflect dietary patterns characterised by more refined carbohydrate sources. Therefore the observed differences between sugar subtypes are likely to reflect underlying dietary patterns rather than the independent effects of individual sugars.

Total fat intake (per 100g) had to be excluded from the adjusted analysis due to strong collinearity with total sugars. Dietary percentage of polyunsaturated fatty acids was associated with a potential protective effect against depression. In 2002, the association was borderline significant (OR: 0.96, 95% CI: 0.91 to 1.00, p = 0.05). By contrast, the 2017 data demonstrated a stronger protective effect (OR: 0.79, 95% CI: 0.75 to 0.83, p < 0.01). Monounsaturated and trans-unsaturated fatty acid associations differed between time points and were inconsistent.

### 3.3 Analysis of change between 2002 and 2017

Linear regression for change in sugars per 100g, with ‘change in HADS’ as the dependent variable shows the adjusted slope coefficient to be 0.63 (95% CI 0.10 to 0.99 p < 0.01) (Table 5). A 100g increase in sugar intake was associated with a 0.63 point increase in HADS score. When ‘change in depression’ (depression sub-score of the HADS questionnaire) is the dependent variable, the adjusted slope coefficient is not statistically significant (b value: 0.25 95% CI -0.02 to 0.48 p = 0.07) (Table 6). Change in the subtypes of sugars and fats were excluded from the analysis due to strong collinearity, and vitamin C was added as a predictor of diet quality which strengthened the association.

**Table 5:** Results of linear regression with ‘change in HADS’ as the dependent variable.

	Unadjusted^a^ B value (95% CI)	P value	Adjusted^b^ B value (95% CI)	P value
Change in Sugars (per 100g)	0.55 (0.10 to 1.00)	0.02	0.63 (0.10 to 0.99)	<0.01

a Unadjusted = adjusted for baseline HADS score.

b Adjusted = adjusted for baseline HADS score, BMI, age, combined IMD, gender, alcohol intake (per 100g) and vitamin C.

**Table 6:** Results of linear regression with ‘change in depression^a^’ as the dependent variable.

	Unadjusted^b^ B value (95% CI)	P value	Adjusted^c^ B value (95% CI)	P value
Change in Sugars (per 100g)	0.22 (−0.03 to 0.47)	0.08	0.25 (−0.02 to 0.48)	0.07

a Change in depression = change in the depression sub-score of the HADS questionnaire.

b Unadjusted = adjusted for baseline Depression score.

c Adjusted = adjusted for baseline HADS score, BMI, age, combined IMD, gender, alcohol intake (per 100g) and vitamin C.

## 4 Discussion

Our findings indicate a significant relationship between elevated sugar intake and poorer mental health outcomes. Longitudinal analyses showed increasing HADS scores correlated with increased sugar consumption, highlighting a potential link. Similarly, cross-sectional analyses revealed that higher sugar intake was associated with greater odds of HADS-defined depression. These findings support an association between higher sugar intake and poorer mental health outcomes.

Regarding sugar subtypes, fructose had the most consistent association with reduced odds of HADS-defined depression in 2002 and 2017. Lactose and sucrose were also associated with lower odds of HADS-defined depression in 2002, and glucose had a strong positive association in 2017 which closely mirrors the findings by Gangwisch et al. [10]. They found that the fifth quintile of glucose intake showed an elevated but non-significant OR for depression incidence, with higher lactose consumption was associated with significantly reduced odds of depression (OR: 0.81, 95% CI 0.72 to 0.92 p < 0.01). These results could be explained by fructose and lactose being lower glycaemic index (GI) than glucose [32].

One of the proposed mechanisms for sugar influencing mood states is the high glycaemic load and thus insulin levels causing a rebound hypoglycaemia, which could affect mood status [33]. Furthermore, fructose is abundant in many fruits [34] which contain high levels of fibre, lowering the GI further. Regular fruit and vegetable consumption has strong associations with improved mental health outcomes lowering the risk of depression [35]. These findings may reflect differences in dietary patterns, glycaemic characteristics and food sources associated with differing sugar subtype compositions and suggest that assessment beyond total sugars alone may be informative. We found that gender, age and BMI all had significant associations with depression at both time points, but the finding for sex should be interpreted with caution due to the small number of males in the cohort (5.9% males in 2002, 9.4% males in 2017).

It is important to note that total sugars and total fats were highly collinear in this cohort, reflecting the fact that many foods high in added sugars also contain substantial amounts of fat. Because of this, fat and sugar subtypes were modelled as percentages rather than absolute intakes. This approach allows their relative contributions to be examined but limits the ability to attribute effects to any single nutrient independent of the others.

In contemporary dietary patterns, however, a substantial proportion of free sugars and combinations of sugar and fat are consumed within ultra-processed foods [36]. These products often contain refined starches, additives and engineered sensory properties that may independently influence mood and eating behaviour [37]. Because our analyses were conducted at the nutrient level, we cannot fully disentangle the effects of sugars per se from the broader ultra-processed food matrix, and it is likely that higher sugar intakes in this cohort also reflect less favourable, ultra-processed dietary patterns. Although dietary fat subtypes were not the primary exposures of interest, some associations were observed within the adjusted models. Higher proportions of polyunsaturated fatty acids were associated with lower odds of HADS-defined depression, which is broadly consistent with previous literature suggesting potential mental health benefits of polyunsaturated fats [[12], [13], [14]]. However, fat subtype findings in this study should be cautiously interpreted because fat variables were primarily included to account for dietary confounding and were highly collinear with total sugar intake.

We showed that with increasing sugar intake, symptoms of anxiety and depression become worse. However, when assessing the depression sub-score of the HADS questionnaire, the slope coefficient was not statistically significant (p = 0.07). This could be due to the participants having relatively low scores with a median depression score of three in 2002 and two at follow-up in 2017. Also, depression sub-score has a much narrower range of values than total HADS (21 compared to 42). These reasons make it difficult to find an association in a population where a small number of people are depressed.

There are a few plausible biological explanations for the link between sugar and depression. A higher hypothalamic-pituitary-adrenal (HPA) axis reactivity leading to dysregulation of the stress response has been indicated as a potential cause of higher depression risk [38]. In addition, high sugar diets could induce a hypoglycaemic effect through an exaggerated insulin response, thereby affecting hormone levels which can influence mood states [39]. It has been suggested that sugar subtypes have differing absorption rates, such as glucose having a faster absorption rate than fructose and other sugars [32]. Therefore, this would theoretically raise blood sugar levels to a greater extent further exaggerating an insulin response. Another proposed mechanism is that diet can influence levels of the growth factor BDNF which has been discussed as enabling hippocampal atrophy in depression [17]. Refined sugar consumption is known to be associated with increased circulatory inflammatory markers which may play a role in mood states [40]. The brain-gut axis theory suggests that a high-sugar diet alters the composition of the gut microbiota, inducing gut dysbiosis which is a reduction in beneficial bacteria and an increase in harmful bacterial species.

Emerging evidence suggests that high-sugar dietary patterns may contribute to gut microbiota dysbiosis, altered inflammatory signalling and disruption of the microbiota–gut–brain axis, which have all been implicated in depressive symptom pathophysiology. Recent reviews have highlighted the potential role of gut microbial metabolites, immune activation and neuroendocrine signalling in mediating diet-related mental health outcomes [41,42]. Obesity could be a catalyst between a higher sugar diet and depression [43], as well as playing a psychological role due to factors such as weight discrimination and bullying [44].

## 5 Strengths

Identifying the effects of a single nutrient in dietary behaviours is extremely difficult as dietary habits are an accumulation of different macro and micronutrients. A strength of this study was adjustment for dietary-related confounders such as the consumption of fat and sugar subtypes. Many sweet and dessert-type foods contain high levels of fats and sugars [15] and fat subtypes have been shown to have strong associations with influencing mental health outcomes [[12], [13], [14]]. Furthermore, breaking down the sugar types gives us an insight into the role sugars play in depression as they each have different characteristics, food sources and absorption rates [32]. Another strength was adjusting for vitamin C in the longitudinal analysis. Vitamin C has been shown to be a useful predictor of diet quality [29], as this can help differentiate which sugars came from fruits and vegetables as opposed to refined sources like sugary beverages and sweets. The temporal order of data collection is an important strength of this study. The FFQ 1 was answered anywhere from 1 to 9 years before the HADS questionnaire and FFQ 3 was answered 3 years before HADS in 2017. This allows assessment of whether dietary habits preceded later depressive symptom outcomes and reduces the likelihood of reverse causation. Combining the results from both cross-sectional and longitudinal analyses reinforced the associations between total sugar intake and depressive symptoms.

## 6 Limitations

We however identified several limitations. The use of FFQs to derive dietary data is a limitation as they are subject to reporting and recall bias which previous evidence has shown to differ by food group, depressive mood and BMI [45,46]. Furthermore, the FFQ data were then used to calculate estimated daily macro and micronutrient intake from frequency and food composition calculations which is a possible source of error. Another limitation with FFQs is that they ask participants to recall dietary habits over a long period of time (1 year), which fails to include daily fluctuations in diet and periods of binge eating or dieting and may predispose to recall and social desirability bias. Despite these limitations, the FFQ used at both time points has been validated against 24-h dietary recalls, weighed food records and certain nutrient biomarkers [47]. After energy adjustment, the dietary nitrogen intake derived from the FFQ positively correlated with urinary nitrogen. Similar trends were observed for potassium, plasma carotenoids, and vitamin C. The FFQ performed similarly to 24-h recalls in placing individuals within the distribution of habitual diet when compared to weighed records, although the recall did not outperform the FFQ [47].

Though we adjusted for several potential confounders, we cannot exclude residual confounding. The strong collinearity between total sugars and total fats restricted the modelling of these nutrients as absolute values, meaning their independent effects cannot be fully separated. Total energy intake was not directly included in the adjusted models but adjustment for BMI, alcohol intake and dietary composition may partially account for broader differences in energy balance and dietary intake.

Sugar intake was modelled per 100 g, which may reduce clinical interpretability but reflects meaningful contrasts in habitual dietary intake within this cohort. Therefore, residual confounding related to total energy intake cannot be excluded. Potentially significant confounders that could have been adjusted for, had the data been available to us, include physical activity, smoking status, antidepressant and other medication use, cardiovascular disease, diabetes and other metabolic or psychiatric comorbidities, recent major life events (e.g., bereavement, divorce, moving house), and broader dietary pattern measures [48]. These conditions may influence both dietary habits and depressive symptom outcomes and be potential sources of residual confounding. Given the relatively low prevalence of HADS-defined depression cases in this cohort, antidepressant use may have been comparatively uncommon, although medication use could still influence both dietary habits and depressive symptom scores.

There are limitations of the TwinsUK cohort. The cohort was predominantly female and largely Caucasian, with older mean ages in analytic samples. As a result, the findings may not be generalisable to men, younger adults, or more ethnically diverse populations. Only 9.4% (2002) and 9.0% (2017) of the population were classed as depression cases (Table 1, Table 2). This is significantly lower than the UK average, as in 2014 19.7% of people in the UK (and 22.5% of females) over 16 years of age, showed symptoms of depression or anxiety [49]. The relatively low prevalence of depression cases in the cohort could be a reason for finding a statistically insignificant slope coefficient for the depression sub-score. In addition, HADS is a validated screening tool rather than a diagnostic psychiatric assessment and may therefore not fully capture clinician-diagnosed depression.

TwinsUK participants only completed the HADS questionnaire at two time points (2002 and 2017). In addition, the interval between dietary assessment and HADS measurement varied between participants, which may have introduced exposure misclassification if dietary habits changed over time. Therefore, reverse causation, whereby low mood influences worsening dietary habits, cannot be completely excluded. Furthermore, the smaller longitudinal sample included only participants with complete repeated dietary and HADS measurements across time points and may therefore be subject to selection bias. However, studies such as the Whitehall II cohort have analysed reverse causation via multiple measures and have suggested that reverse causation alone is unlikely to fully explain observed associations [9]. Finally, using estimated daily nutrient intakes does not take into account the glycaemic load (GL) of certain foods. Any influence that sugars have on mood is likely commensurate with the proportion they constitute in the overall diet, so a limitation is that we only examined total sugars and percentage of sub-sugars rather than dietary GI and GL in the diet. We are aware of several prior studies and a meta-analysis that examined the relationship between GI and GL with depression [10,[50], [51], [52]]. One meta-analysis found a positive association between GI and GL with risk of depression [52].

We did not classify foods according to level of processing (for example using NOVA categories), so we were unable to examine whether ultra-processed foods, which are a major source of combined sugar and fat and food additives, specifically mediated the associations observed. This limited our ability to separate the effects of sugars from those of the ultra-processed food matrix, and some residual confounding by overall dietary pattern is likely. Finally, although TwinsUK provides a unique opportunity to perform within-pair analyses in monozygotic twins, this was not feasible to conduct. Our analyses did not explicitly account for clustering within twin pairs, which may influence precision of effect estimates and standard errors. The subset of identical twin pairs with complete dietary and HADS data across the relevant time points was substantially smaller than the full analytic cohort, which may have limited statistical power and precision of effect estimates, particularly for longitudinal analyses.

## 7 Implications for practice and future research directions

Our findings can inform discussion about the role of diet, particularly sugars, in the management of depression in adults. Diet and nutrition, including sugar intake, may be relevant considerations in broader strategies aimed at supporting mental health and reducing depressive symptoms. Future research should focus on understanding the efficacy and dose-response relationship of foods and dietary patterns on depression. There is a crucial need to better educate the public and medical professionals about the role of diet and nutrients in sustaining mental health and providing better mental health outcomes. Future large-scale studies and randomised controlled trials (RCT) are needed to further assess the effects of dietary interventions on depressive symptoms and mental health outcomes. Co-twin control analyses could help further account for shared genetic and early environmental confounding and represents an important direction for future research.

## 8 Conclusion

These results suggest that high sugar intake was associated with greater odds of HADS-defined depression in Caucasian adult women. Although whole foods and low sugar diets have been shown to be beneficial for depressive symptoms in cohort studies and in RCTs, further RCTs should be undertaken to specifically assess if diets rich in whole and low sugar foods, such as wholegrains and legumes, could serve as supportive approaches for depressive symptoms in adults.

## Financial Support

FM, Doctoral Fellowship (300957), was funded by the National Institute for Health and Care Research (NIHR) from 2021 to 25, and is now funded by a Pre-Application Support Fund (508051). TwinsUK is funded by the Wellcome Trust, Medical Research Council, Versus Arthritis, European Union Horizon 2020, Chronic Disease Research Foundation (CDRF), Wellcome Leap Dynamic Resilience Programme (co-funded by Temasek Trust), Zoe Ltd, NIHR Clinical Research Network and Biomedical Research Centre based at Guy's and St Thomas' NHS Foundation Trust in partnership with King's College London. None of the other authors received financial support for this manuscript. The views expressed are those of the authors and not necessarily those of the NIHR or the Department of Health and Social Care.

## CRediT authorship contribution statement

**Idrees Mughal:** Conceptualization, Data curation, Formal analysis, Investigation, Methodology, Visualization, Writing – original draft, Writing – review & editing. **Zeeshan Mughal:** Writing – review & editing. **Khaylen Mistry:** Data curation, Writing – review & editing. **Noor Dhakal:** Data curation. **Faraz Mughal:** Funding acquisition, Supervision, Writing – review & editing.

## Declaration of competing interest

The authors have no relevant financial or non-financial interests to disclose.

## Data Availability

The authors do not have permission to share data.
